# Supplementary material for: Aβ43‐producing PS1 FAD mutants cause altered substrate interactions and respond to γ‐secretase modulation
Source: EMBO Rep. 2019 Nov 25;21(1):e47996. doi: 10.15252/embr.201947996 (PMC6945062; doi:10.15252/embr.201947996)
Supplement: Supplementary file 1 — Appendix [file EMBR-21-e47996-s001.pdf]

Appendix

Appendix Figure S1 - Species-specific comparison of Aβ secreted by Aβ43-generating PS1 FAD mutants — Page 2

Appendix Figure S2 - Location of FAD mutations and interacting substrate residues in the C83–PS1 γ-secretase complex structure — Page 3

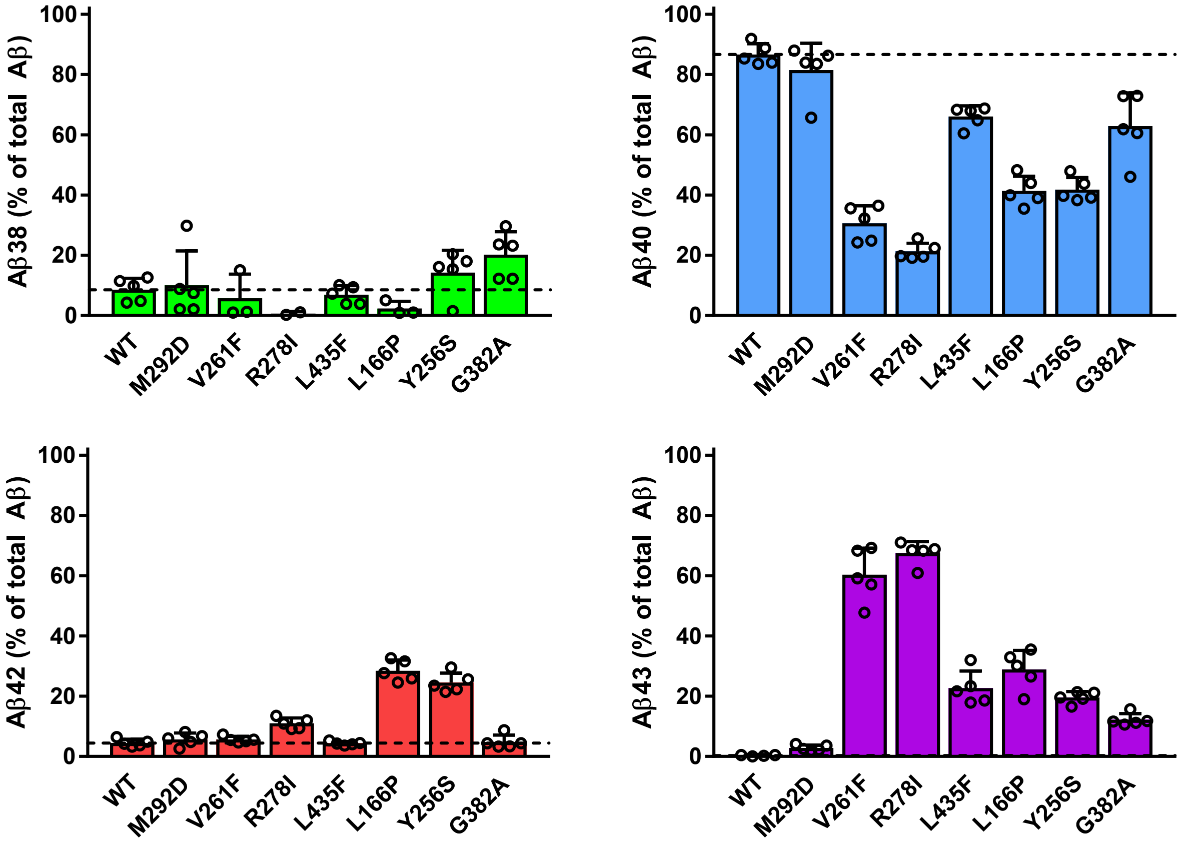

Appendix Figure S1. Species-specific comparison of Aβ secreted by Aβ43-generating PS1 FAD mutants

Analysis of conditioned medium of HEK293/sw cells overexpressing WT or mutant PS1 by Aβ species-specific ELISA. Shown are the ratios of the different Aβ species compared to total Aβ (Aβ38 +Aβ40 + Aβ42 + Aβ43) measured. The dashed line represents the levels of PS1 WT secretion (n = 5 biological replicates). Data are presented as mean ±SD).

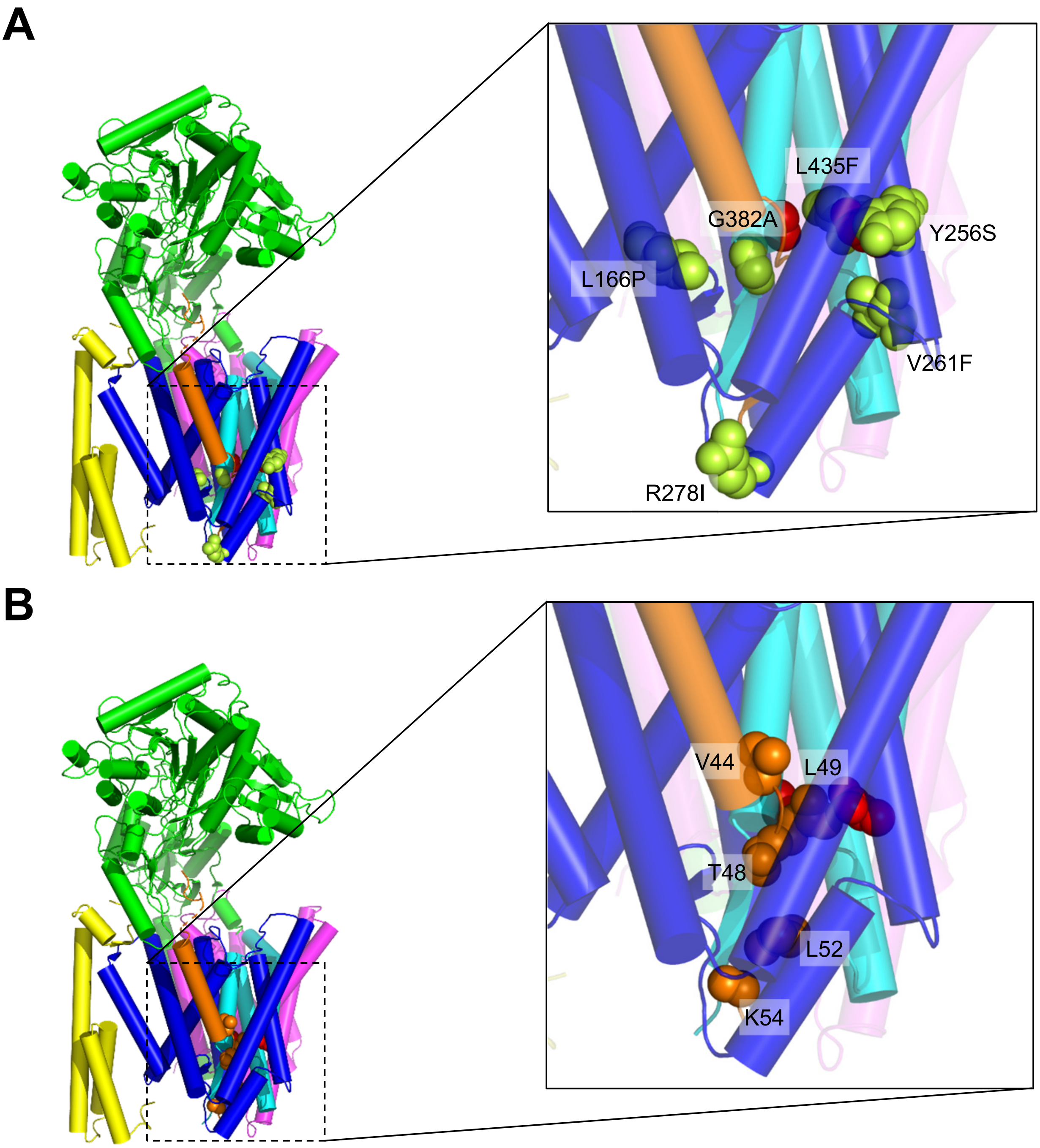

Appendix Figure S2. Location of FAD mutations and interacting substrate residues in the C83–PS1 γ-secretase complex structure.

(A and B) Residues of presenilin FAD mutants (A) and C99-PS1 crosslinking residues (B) are highlighted in the substrate–enzyme complex structure (PDB: 6IYC). Side chains of FAD mutant residues (A) are displayed as lime green, interacting substrate residues (B) as orange and catalytic aspartate residues (A and B) as red spheres respectively. Color coding: C83, orange; PS1 NTF, blue; PS1 CTF, cyan, NCT, green, PEN-2, yellow; APH-1, magenta.
