# Supplementary material for: Aβ43‐producing PS1 FAD mutants cause altered substrate interactions and respond to γ‐secretase modulation
Source: EMBO Rep. 2019 Nov 25;21(1):e47996. doi: 10.15252/embr.201947996 (PMC6945062; doi:10.15252/embr.201947996)
Supplement: Supplementary file 2 — Expanded View Figures PDF [file EMBR-21-e47996-s002.pdf]

Expanded View Figures

Figure EV1. Modulation of selected PS1 FAD mutants with different classes of GSMs.

A Structure of GSM-1.

B Structure of RO-02.

C, D ELISA of Aβ species in conditioned media of HEK293/sw cells overexpressing WT or PS1 L166P and R278I mutants treated with GSM-1 (C), RO-02 (D), or vehicle (DMSO) showing the secretion of Aβ38, Aβ40, Aβ42, and Aβ43 compared to total Aβ (Aβ38 + Aβ40 + Aβ42 + Aβ43) (n = 2 biological replicates). Data are presented as mean.

Source data are available online for this figure.

© 2019 The Authors

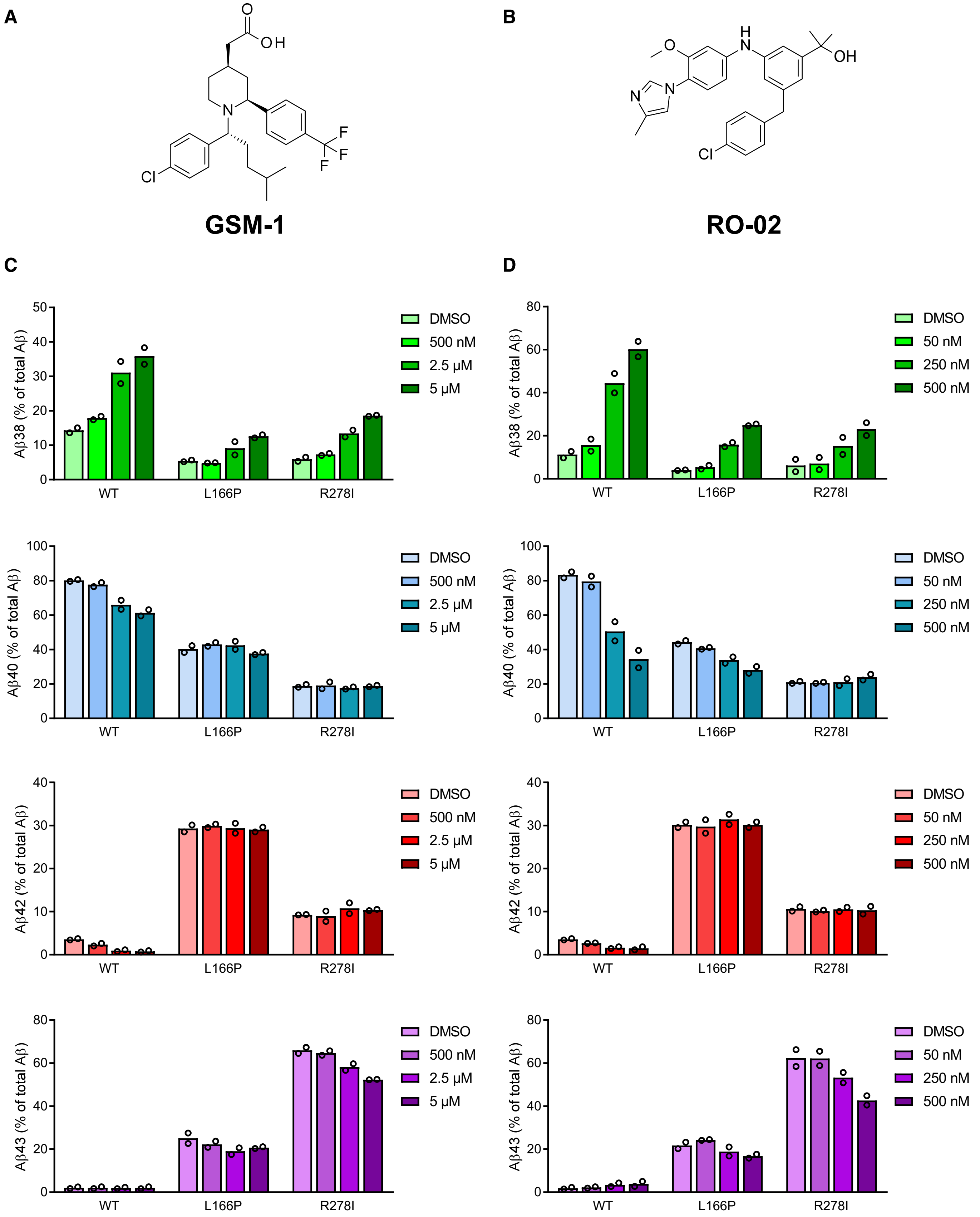
A
B
O
OH
N
F
F
F
Cl
GSM-1
H
N
O
OH
N
N
Cl
RO-02
C
D
Aβ38 (% of total Aβ)
Aβ40 (% of total Aβ)
Aβ42 (% of total Aβ)
Aβ43 (% of total Aβ)
WT
L166P
R278I
DMSO
500 nM
2.5 µM
5 µM
DMSO
50 nM
250 nM
500 nM

Figure EV1.

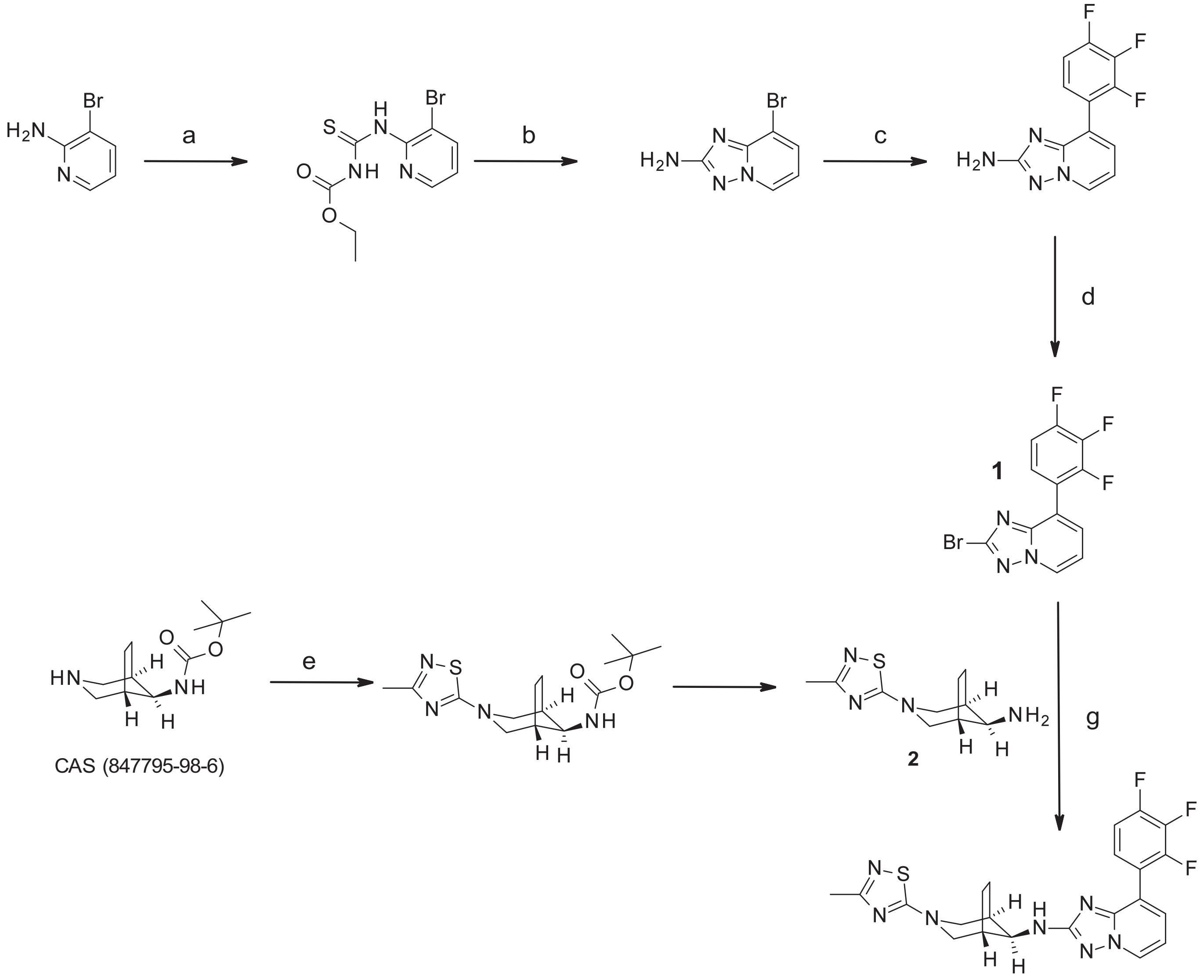

Figure EV2. Synthesis of RO7019009.

Schematic overview of the steps of the RO7019009 synthesis. Reagents and conditions: a) EtO_2CNCS (1.1 equiv), dioxane, room temperature, 3 h, quant.; b) $NH_2OH{\cdot}HCl$ (5 equiv), i-Pr_2NEt (3 equiv), MeOH/EtOH (1/1), room temperature to 60°C, 4 h, 94%; c) 2,3,4-trifluorophenylboronic acid (1.3 equiv), Cs_2CO_3 (2 equiv), $PdCl_2(DPPF){\cdot}CH_2Cl_2$ (0.1 equiv), dioxane/H_2O (10/1), 100°C, overnight, 82%; d) tert-butyl nitrite (1.5 equiv), Cu(II)Br_2 (1.5 equiv), acetonitrile, 75°C, 2 h, 87%; e) 5-chloro-3-methyl-[1,2,4] thiadiazole (1.2 equiv), Et_3N (1.5 equiv), EtOH, 80°C, overnight, 97%; f) TFA (10 equiv), CH_2Cl_2, room temperature, overnight, 94%; g) intermediate 2 (1 equiv), sodium phenoxide (1.6 equiv), xantphos (0.16 equiv), $Pd_2dba{\cdot}CHCl_3$ (0.08 equiv), 145°C, 45 min in microwave, 57%.

© 2019 The Authors

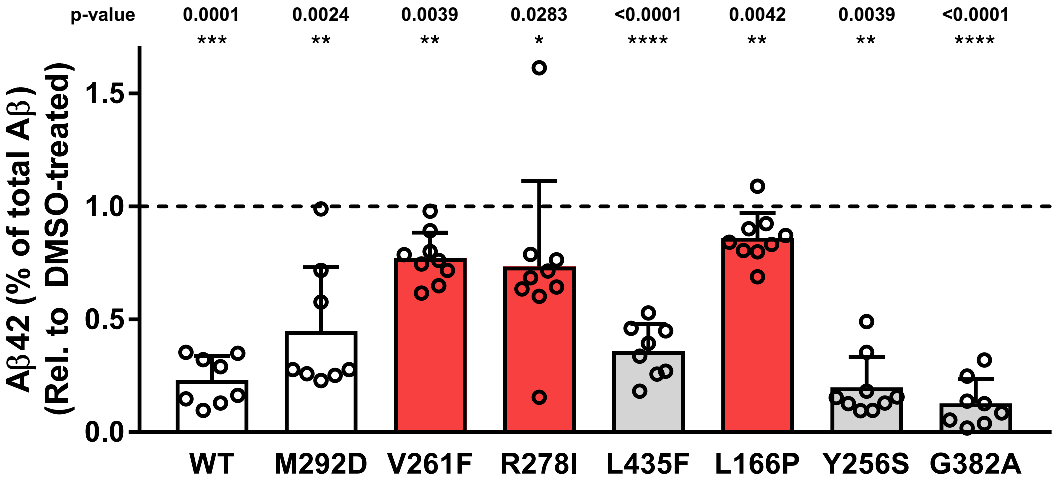

Figure EV3. Aβ42-lowering effects of RO7019009 on refractory PS1 FAD mutants.

ELISA of Aβ species in conditioned media of HEK293/sw cells overexpressing WT or mutant PS1 treated with 500 nM RO7019009 or vehicle (DMSO) showing the secretion of Aβ42 compared to total Aβ (Aβ38 + Aβ40 + Aβ42 + Aβ43) ($n = 9$; including data taken from Fig 4C ($n = 5$) and 4 additional experiments, data are presented as mean ± SD). Statistical significance was tested using paired t-test. In case of the V261F and the Y256S mutant, the data were not normally distributed and were therefore assessed using Wilcoxon matched-pairs signed rank test.

Source data are available online for this figure.

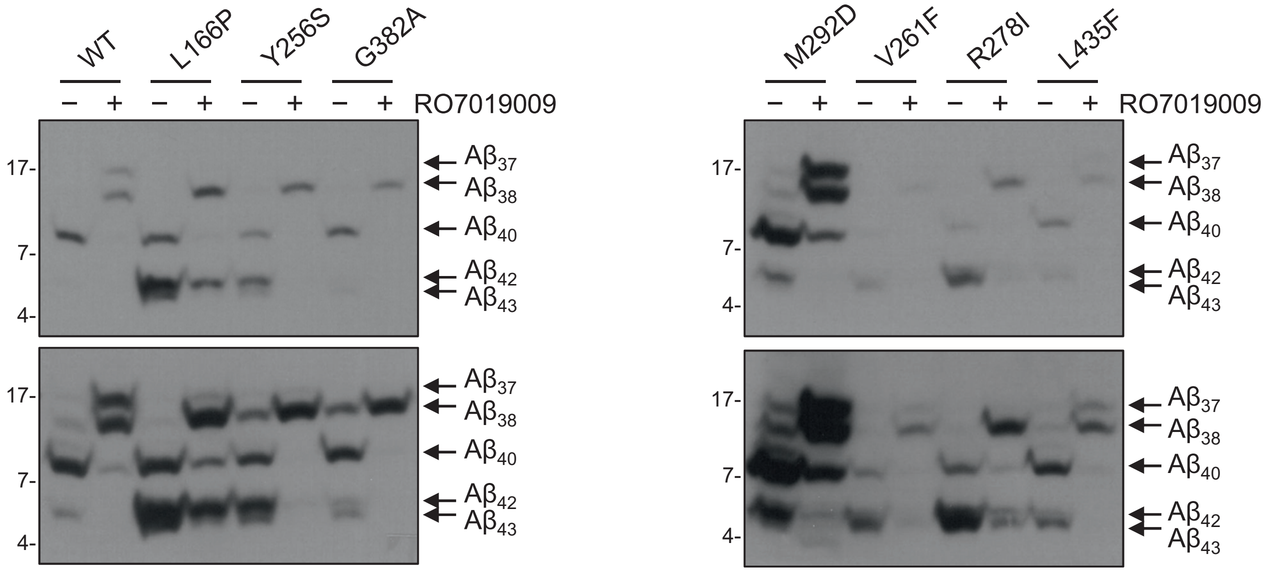

Figure EV4. Modulation of Aβ profiles of PS1 FAD mutants by RO7019009.

Immunoblot analysis of Aβ species in conditioned media of HEK293/sw cells overexpressing WT or mutant PS1 treated with 500 nM RO7019009 or vehicle (DMSO) after separation by Tris–Bicine urea SDS–PAGE.

Source data are available online for this figure.

© 2019 The Authors

Figure EV5. RO7019009 maintains activity toward CHAPSO-solubilized γ-secretase.

A Sandwich immunoassay of short Aβ species (Aβ37 + Aβ38) generation from recombinant APP C100-His_6 substrate by CHAPSO-solubilized γ-secretase of HEK293/sw cells overexpressing WT or mutant PS1 in the presence of RO7019009 or vehicle (DMSO) (n = 3 biological replicates).

B Immunoblot analysis of AICD generation from recombinant APP C100-His_6 substrate by CHAPSO-solubilized γ-secretase of HEK293/sw cells overexpressing WT or mutant PS1 in the presence of RO7019009, L-685,458 (5 μM), or vehicle (DMSO).

C Quantitation of AICD generation in (B) (n = 4–5 biological replicates).

Data information: Data in (A) and (C) are presented as mean ± SD.
Source data are available online for this figure.

© 2019 The Authors

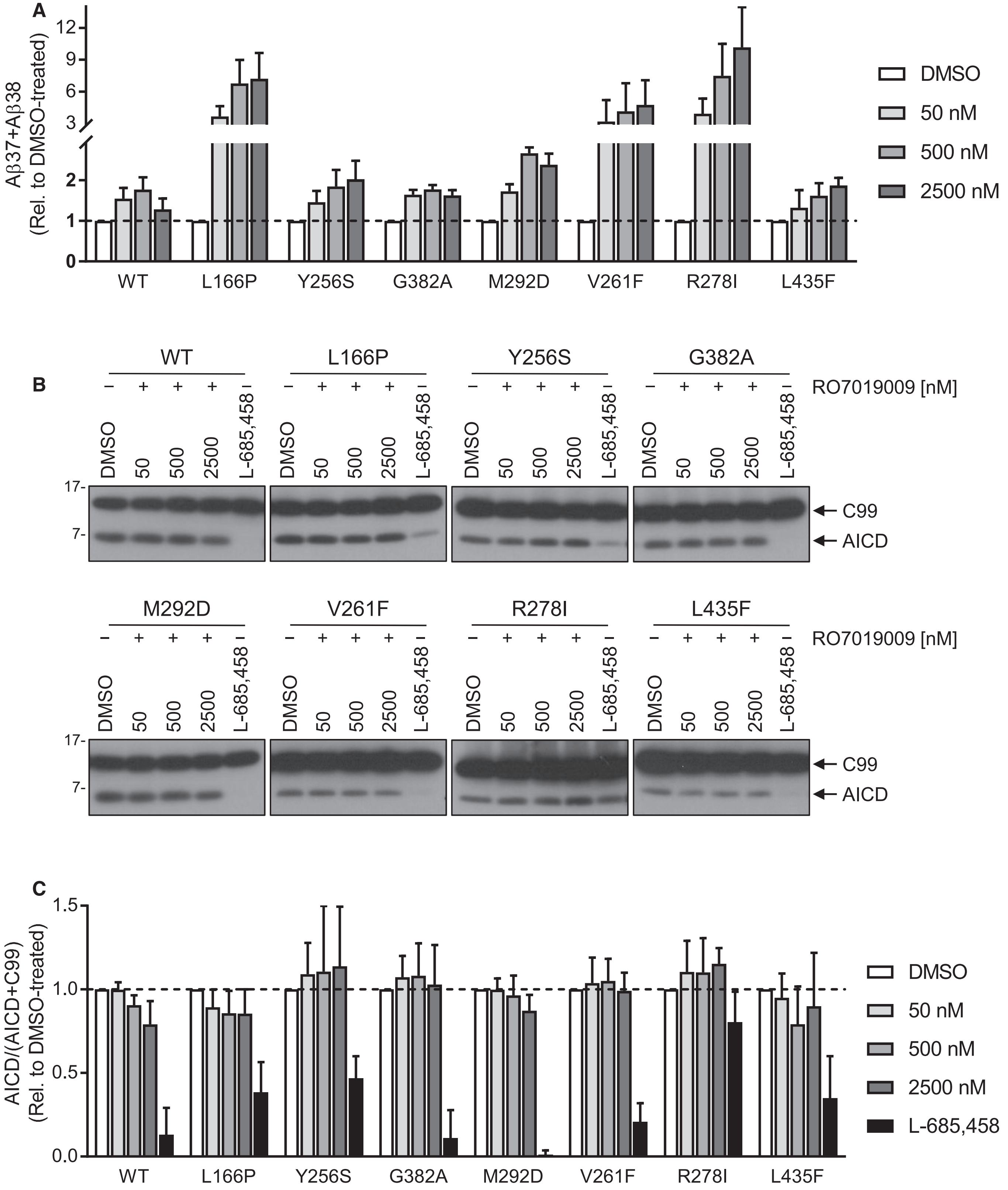
A
Aβ37+Aβ38
(Rel. to DMSO-treated)
12
9
6
3
2
1
0
WT
L166P
Y256S
G382A
M292D
V261F
R278I
L435F
DMSO
50 nM
500 nM
2500 nM
B
RO7019009 [nM]
DMSO
50
500
2500
L-685,458
17-
7-
C99
AICD
C
AICD/(AICD+C99)
(Rel. to DMSO-treated)
1.5
1.0
0.5
0.0
L-685,458

Figure EV5.
