# Supplementary material for: Aβ43‐producing PS1 FAD mutants cause altered substrate interactions and respond to γ‐secretase modulation
Source: EMBO Rep. 2019 Nov 25;21(1):e47996. doi: 10.15252/embr.201947996 (PMC6945062; doi:10.15252/embr.201947996)
Supplement: Supplementary file 3 — Table EV1 [file EMBR-21-e47996-s003.docx]

**Table EV1. RO7019009 key physicochemical and pharmacokinetic properties predicting CNS drug-likeness**

| Solubility (μg/ml) | LogD^a^ | PAMPA^b^ (permeability) | CLmicr (h/m)^c^  (μl/min/mg) |
| --- | --- | --- | --- |
| 3 | 4 | High | <10/<10 (low/low) |

*^a^LogD= logarithm of the octanol-water distribution coefficient at pH 7.4; ^b^PAMPA: Parallel artificial membrane parallel assay; ^c^CLmicr (h/m): human/mouse microsomal clearance*
