# Supplementary material for: Aβ43‐producing PS1 FAD mutants cause altered substrate interactions and respond to γ‐secretase modulation
Source: EMBO Rep. 2019 Nov 25;21(1):e47996. doi: 10.15252/embr.201947996 (PMC6945062; doi:10.15252/embr.201947996)
Supplement: Supplementary file 4 — Source Data for Expanded View and Appendix [file EMBR-21-e47996-s009.zip › Appendix_Fig_S1_source.pdf]

Aβx (% of total Aβ)

	Aβ38					Aβ40					Aβ42					Aβ43				
WT	9.8	11.4	12.6	4.8	4.3	85.3	83.9	83.5	88.8	91.8	4.9	4.2	3.3	6.4	3.7	n.d.	0.5	0.5	0	0.2
M292D	7.4	29.8	8.8	2.1	2.1	83.5	65.7	83.9	86.2	87.9	6.8	2.6	4.8	8.1	6	2.2	1.9	2.5	3.6	4.1
V261F	15	n.d.	n.d.	1.2	1	32.2	36.4	35.6	24.9	24.3	5.1	4.6	7.3	5.6	5.5	47.7	59.1	57.1	68.3	69.2
R278I	n.d.	n.d.	n.d.	1.1	0.2	25.6	22.4	19.2	19.5	19.7	13.5	9.4	12	10.9	9.1	60.9	68.3	68.8	68.5	71
L435F	7.3	9.4	10.1	3.9	3.9	68.7	64.8	68	68.3	60.5	5.3	4.3	4	4.5	3.6	18.6	21.6	17.9	23.3	32
L166P	5.1	n.d.	n.d.	0.9	1	48.2	44	35.5	39.9	39	27.7	25.9	31.6	32.6	24.5	19	30.1	32.9	26.6	35.5
Y256S	20.4	18	16.1	1.5	15.3	39.7	38.3	39.1	47.9	43.7	23.5	22.3	25.6	29.5	21.5	16.5	21.4	19.2	21.1	19.6
G382A	29.6	23.6	23.2	12.2	12.2	46	61.8	60.6	72.8	72.9	8.7	3.3	4.4	4.4	3.4	15.7	11.2	11.8	10.6	11.6

Source Data Appendix Figure S1
