# Supplementary material for: Aβ43‐producing PS1 FAD mutants cause altered substrate interactions and respond to γ‐secretase modulation
Source: EMBO Rep. 2019 Nov 25;21(1):e47996. doi: 10.15252/embr.201947996 (PMC6945062; doi:10.15252/embr.201947996)
Supplement: Supplementary file 4 — Source Data for Expanded View and Appendix [file EMBR-21-e47996-s009.zip › Fig_EV1_source.pdf]

C Aβ species (% of total Aβ) (GSM-1)

Aβ38	DMSO		500 nM		2500 nM		5000 nM	
WT	15	13.6	17.4	18.4	34.3	27.9	38.3	33.5
L166P	5.2	5.7	4.9	4.8	11	7.2	13	12.1
R278I	5.3	6.5	7	7.6	14.4	12.4	18.7	18.4

Aβ40	DMSO		500 nM		2500 nM		5000 nM	
WT	79.6	80.6	78.9	76.5	63.4	68.6	59.5	63.1
L166P	42.2	38.3	41.9	44	40.1	44.8	37	38.3
R278I	18.1	19.7	17.2	21.1	17	18.3	18.4	19.2

Aβ42	DMSO		500 nM		2500 nM		5000 nM	
WT	3.7	3.4	2	2.6	0.7	1.1	0.6	0.9
L166P	30.1	28.5	29.5	30.3	28.2	30.5	29.6	28.5
R278I	9.3	9.2	10.1	7.7	12	9.5	10.5	10.2

Aβ43	DMSO		500 nM		2500 nM		5000 nM	
WT	1.7	2.4	1.7	2.5	1.6	2.3	1.6	2.5
L166P	22.6	27.5	23.7	20.8	20.6	17.5	20.3	21.1
R278I	67.3	64.5	65.7	63.6	56.6	59.7	52.4	52.2

D Aβ species (% of total Aβ) (RO-02)

Aβ38	DMSO		50 nM		250 nM		500 nM	
WT	9.7	12.7	12.8	18.4	39.9	48.9	56.7	63.8
L166P	4.1	3.8	4.5	6.2	16.8	14.9	25.4	24.6
R278I	3.3	9	4.2	9.9	11.3	19.2	20.1	26

Aβ40	DMSO		50 nM		250 nM		500 nM	
WT	85.1	81.6	82.6	76.4	56.1	45	39.5	29.4
L166P	43.2	45.2	40.2	41.2	32	35.7	26.2	30.1
R278I	20.4	21.5	20.4	21	23	19	25.6	22.3

Aβ42	DMSO		50 nM		250 nM		500 nM	
WT	3.7	3.4	2.7	2.6	1.4	1.8	1.1	1.8
L166P	29.5	30.8	31.3	28.2	30.2	32.6	30.8	29.5
R278I	10.1	11.1	9.9	10.3	10	11	9.4	11.2

Aβ43	DMSO		50 nM		250 nM		500 nM	
WT	1.5	2.2	1.9	2.5	2.6	4.3	2.7	5
L166P	23.2	20.2	24.1	24.4	21	16.8	17.6	15.8
R278I	66.2	58.4	65.5	58.8	55.6	50.8	44.8	40.5

Source Data Figure EV1
