# Supplementary material for: Aβ43‐producing PS1 FAD mutants cause altered substrate interactions and respond to γ‐secretase modulation
Source: EMBO Rep. 2019 Nov 25;21(1):e47996. doi: 10.15252/embr.201947996 (PMC6945062; doi:10.15252/embr.201947996)
Supplement: Supplementary file 4 — Source Data for Expanded View and Appendix [file EMBR-21-e47996-s009.zip › Fig_EV3_source.pdf]

Aβ42 at 500 nM RO7019009

	Aβ42 (% of total Aβ; Rel. to DMSO-treated)									Statistical test	p-value
WT	0.35	n.d.	0.29	0.35	0.13	0.16	0.10	0.15	0.32	Student's paired t-test	0.0001
M292D	0.99	0.58	0.72	0.28	n.d.	0.26	0.28	0.25	0.23	Student's paired t-test	0.0024
V261F	0.98	0.79	0.62	0.72	0.80	0.89	0.76	0.65	0.74	Wilcoxon matched-pairs signed rank test	0.0039
R278I	1.61	0.79	0.68	0.76	0.60	0.63	0.64	0.71	0.15	Student's paired t-test	0.0283
L435F	0.45	0.53	n.d.	0.39	0.26	0.34	0.18	0.27	0.46	Student's paired t-test	<0.0001
L166P	0.92	0.83	0.90	0.84	0.80	0.69	0.87	1.09	0.80	Student's paired t-test	0.0042
Y256S	0.35	0.18	0.10	0.16	0.49	0.15	0.10	0.13	0.13	Wilcoxon matched-pairs signed rank test	0.0039
G382A	0.25	0.14	0.13	0.32	n.d.	0.04	0.09	0.05	0.02	Student's paired t-test	<0.0001

Source Data Figure EV3
