# Supplementary material for: Aβ43‐producing PS1 FAD mutants cause altered substrate interactions and respond to γ‐secretase modulation
Source: EMBO Rep. 2019 Nov 25;21(1):e47996. doi: 10.15252/embr.201947996 (PMC6945062; doi:10.15252/embr.201947996)
Supplement: Supplementary file 4 — Source Data for Expanded View and Appendix [file EMBR-21-e47996-s009.zip › Fig_EV5_source.pdf]

A Aβ37+Aβ38 production (Rel. to DMSO-treated)

	50 nM RO7019009			500 nM RO7019009			2500 nM RO7019009		
WT	1.24	1.76	1.62	1.42	1.96	1.92	0.97	1.42	1.45
L166P	4.21	2.55	4.24	8.62	4.29	7.37	9.73	4.89	7.01
Y256S	1.73	1.17	1.47	2.31	1.58	1.62	2.50	1.58	1.97
G382A	1.70	1.72	1.51	1.87	1.78	1.65	1.60	1.50	1.77
M292D	1.75	1.54	1.89	2.53	2.65	2.81	2.34	2.12	2.66
V261F	n.d.	4.62	1.74	n.d.	6.01	2.26	n.d.	6.38	3.11
R278I	2.34	4.28	5.12	4.10	8.59	9.78	6.44	10.00	14.03
L435F	1.08	1.82	1.07	1.41	1.97	1.46	1.65	2.00	1.95

B

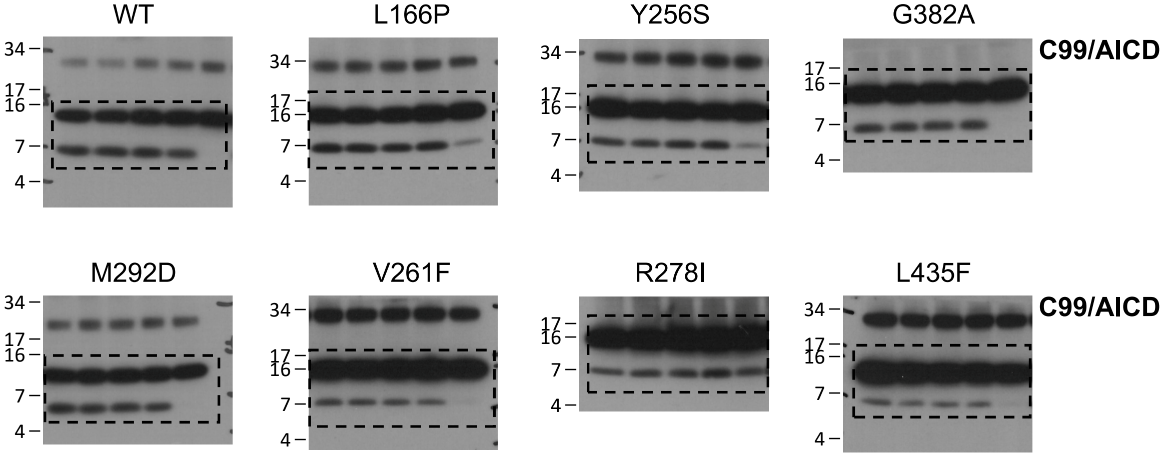

C AICD production (Rel. to DMSO-treated)

	50 nM RO7019009					500 nM RO7019009					2500 nM RO7019009					5 µM L-685.458				
WT	1.06	1.00	0.99	1.01	0.93	0.84	0.99	0.86	0.91	0.93	0.69	1.00	0.64	0.81	0.81	0.38	0.21	0.02	0.04	0.00
L166P	0.95	0.75	0.85	1.03	0.88	0.96	1.01	0.68	0.86	0.77	1.01	0.92	0.71	0.94	0.68		0.38	0.37	0.61	0.16
Y256S	1.29	1.15	0.79	1.12	1.10	1.29	1.57	0.52	0.96	1.19	1.24	1.35	0.60	0.99	1.51		0.65	0.36	0.47	0.39
G382A	1.28	1.12	0.98	1.00	0.99	1.26	1.33	0.92	0.95	0.96	1.33	1.21	0.91	0.74	0.96		0.31	-0.11	0.13	0.11
M292D	1.08	1.03	0.91	0.95	1.02	1.10	1.07	0.87	0.83	0.95	0.92	0.96	0.77	0.77	0.94		0.04	0.00	0.02	-0.02
V261F	1.03	n.d.	0.93	1.26	0.94	1.08	n.d.	0.91	1.22	0.99	1.15	n.d.	0.92	0.98	0.91		n.d.	0.18	0.33	0.11
R278I	1.35	n.d.	1.13	0.93	1.00	1.39	n.d.	0.97	0.93	1.11	1.17	n.d.	1.06	1.10	1.28		n.d.	0.64	0.77	1.00
L435F	0.81	1.02	1.16	0.93	0.83	0.56	0.69	1.16	0.77	0.78	0.82	1.13	1.33	0.57	0.65		0.71	0.32	0.17	0.19

Source Data Figure EV5
