# Supplementary figures and images for: Aβ43‐producing PS1 FAD mutants cause altered substrate interactions and respond to γ‐secretase modulation
Source: EMBO Rep. 2019 Nov 25;21(1):e47996. doi: 10.15252/embr.201947996 (PMC6945062; doi:10.15252/embr.201947996)

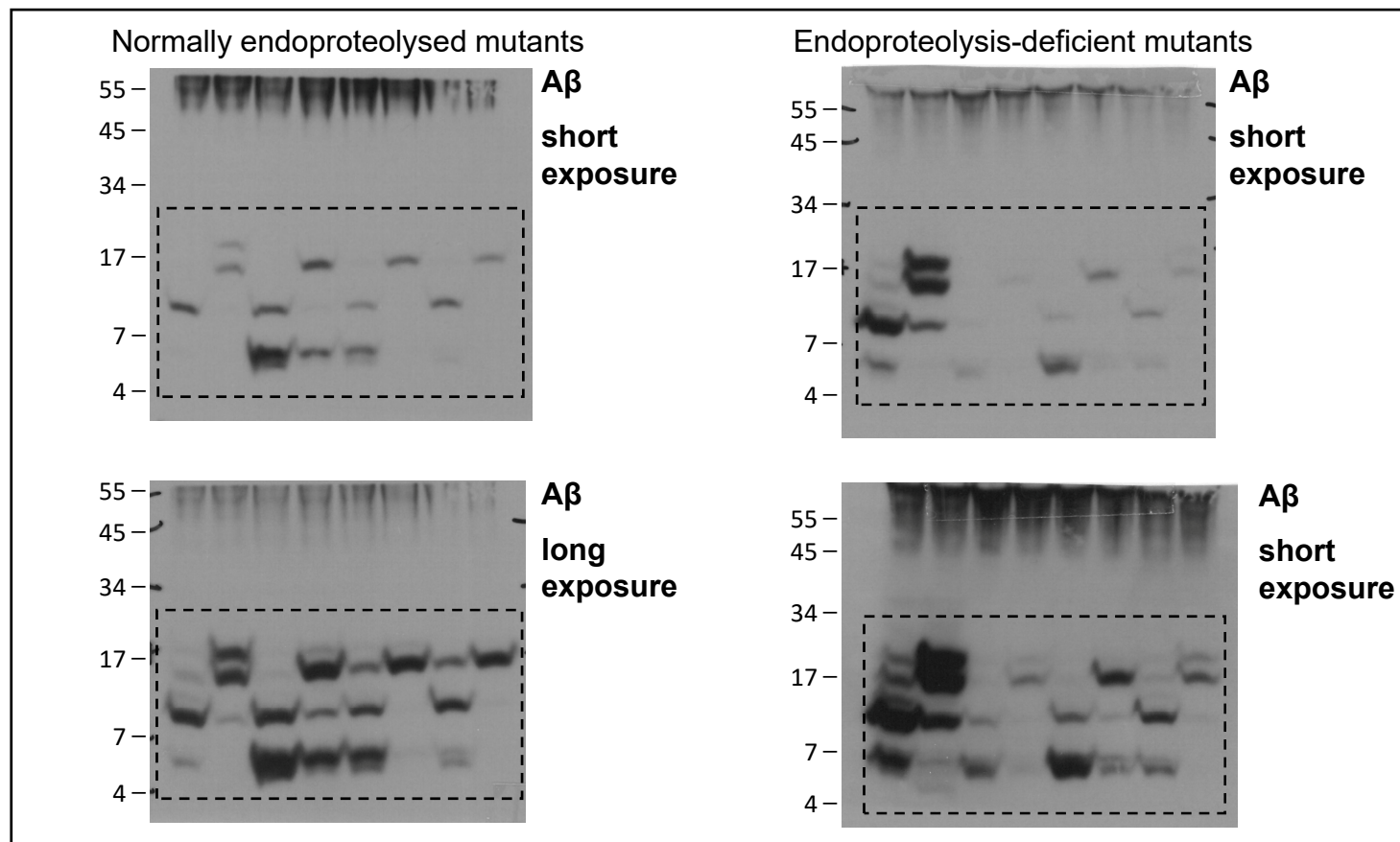

Supplement: Supplementary file 4 — Source Data for Expanded View and Appendix [file EMBR-21-e47996-s009.zip › Fig_EV4_source.pdf]

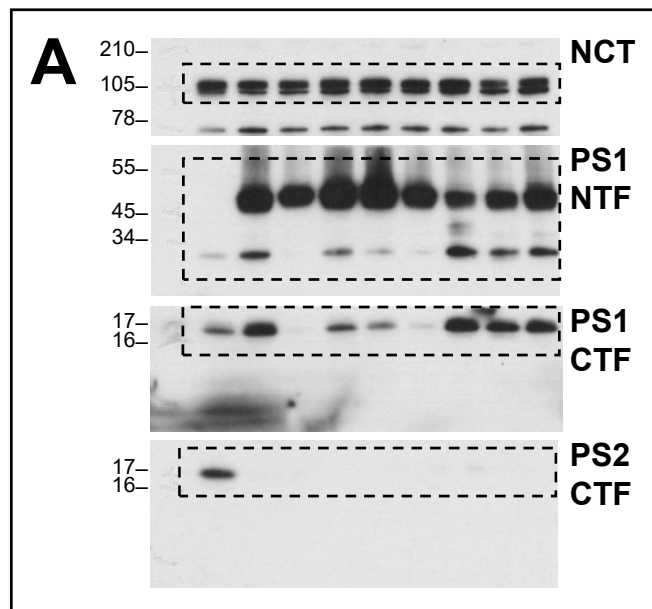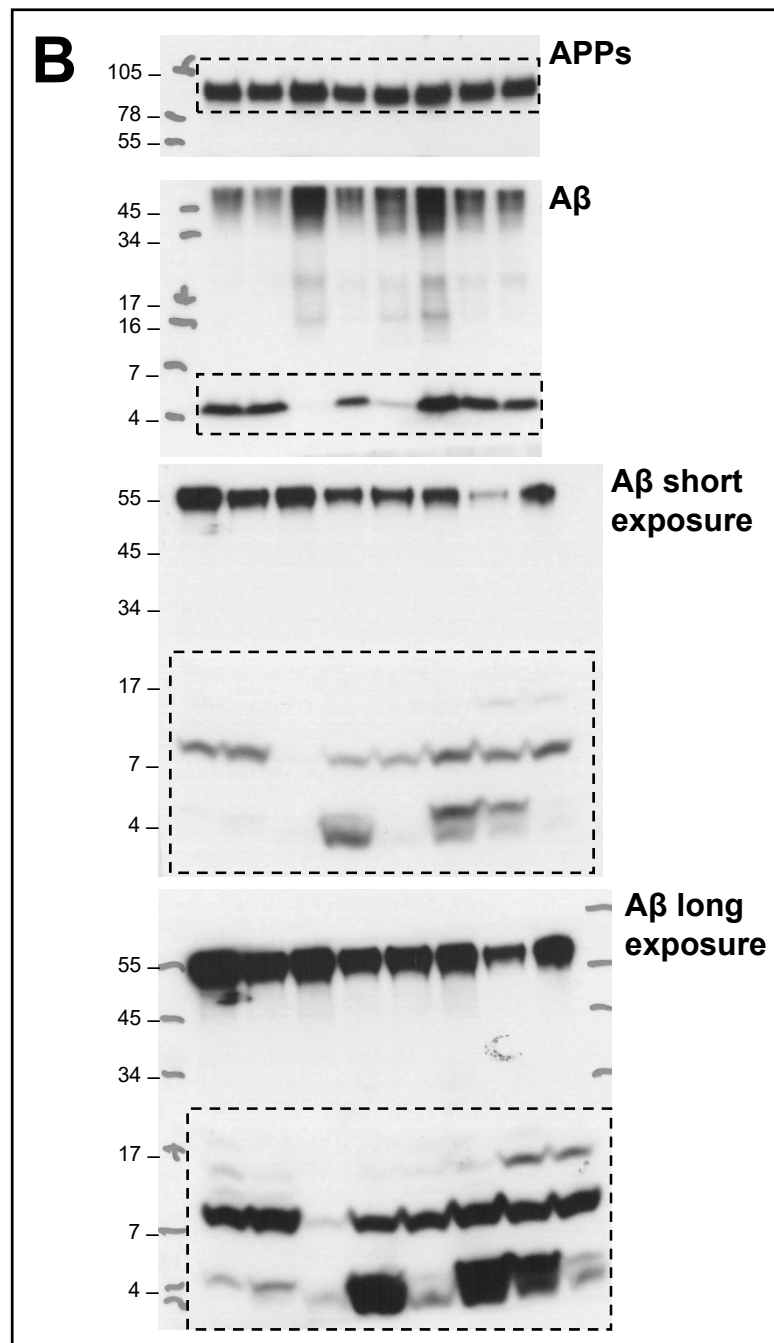

Source Data Figure 1

Supplement: Supplementary file 6 — Source Data for Figure 1 [file EMBR-21-e47996-s004.pdf]
