# Supplementary material for: Aβ43‐producing PS1 FAD mutants cause altered substrate interactions and respond to γ‐secretase modulation
Source: EMBO Rep. 2019 Nov 25;21(1):e47996. doi: 10.15252/embr.201947996 (PMC6945062; doi:10.15252/embr.201947996)
Supplement: Supplementary file 7 — Source Data for Figure 2 [file EMBR-21-e47996-s005.pdf]

A

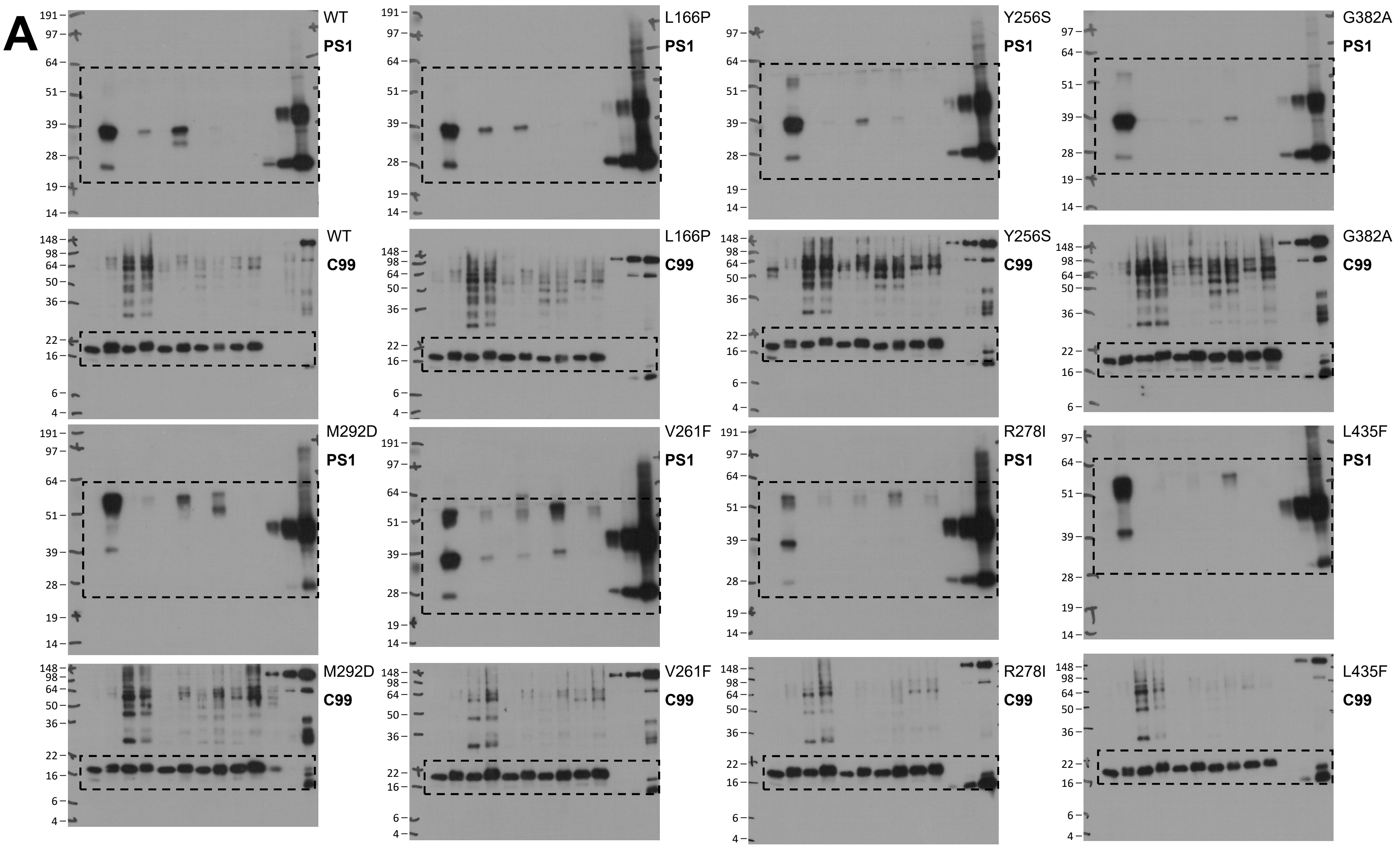

B

	CL efficiency T48/L49 (Change from CTRL) [lg]				
WT	0	0	0	0	0
L166P	1.053	0.620	0.423	0.915	0.673
Y256S	0.019	-0.393	-0.338	0.100	-0.710
G382A	-0.035	0.174	0.378	0.369	
M292D	0	0	0	0	
V261F	0.352	0.568	1.137	0.001	
R278I	0.171	0.224	0.239	0.656	
L435F	0.797	0.476	0.956	0.381	

C

	CL efficiency K54/V44 (Change from CTRL) [lg]				
WT	0	0	0	0	0
L166P	0.420	0.451	0.673	0.641	0.428
Y256S	0.132	-0.256	0.346	-0.159	0.066
G382A	-0.134	-0.309	-0.102	-0.264	
M292D	0	0	0	0	
V261F	0.460	0.286	0.857	0.141	
R278I	0.576	0.768	1.120	0.523	
L435F	-0.058	0.306	0.850	-0.105	

Source Data Figure 2
