# Supplementary material for: Aβ43‐producing PS1 FAD mutants cause altered substrate interactions and respond to γ‐secretase modulation
Source: EMBO Rep. 2019 Nov 25;21(1):e47996. doi: 10.15252/embr.201947996 (PMC6945062; doi:10.15252/embr.201947996)
Supplement: Supplementary file 8 — Source Data for Figure 3 [file EMBR-21-e47996-s006.pdf]

B

RO7019009	Aβ42 (Rel. to DMSO-treated)				Aβ40 (Rel. to DMSO-treated)				Aβ38 (Rel. to DMSO-treated)			
DMSO	1	1	1	1	1	1	1	1	1	1	1	1
50 nM	0.51	0.34	0.31	0.50	0.83	0.61	0.64	0.72	2.64	3.83	3.67	3.08
250 nM	0.41	0.14	0.23	0.23	0.62	0.26	0.24	0.36	4.48	6.25	6.35	5.61
500 nM	0.22	0.08	0.16	0.22	0.31	0.18	0.13	0.34	7.34	6.75	7.05	5.80

C

RO7019009 [lg(M)]	Aβ38 (% from vehicle-treated)			Aβ40 (% from vehicle-treated)			Aβ42 (% from vehicle-treated)		
-10.3345	98.9	100.5	102.2	91.5	99.7	102.5	91.3	100	102.7
-9.5563	108.7	106.3	110	95.8	97.1	106.5	90.9	95.5	101.3
-8.77815	125.1	129.7	130.2	96.4	94.8	112.2	83.9	82.5	96.4
-8	183.4	188.8	191	76.3	62.1	83.2	54.7	49.3	67.5
-7.22185	264	229.7	312.9	37.3	29.8	39.2	19.2	20.6	27.7
-6.4437	374.8	383.3	449.7	12.8	10.8	13.1	2.1	n.d.	n.d.
-5.66555	389.7	545	425.2	4.6	4.1	4.5	n.d.	n.d.	n.d.

D

RO7019009 [lg(M)]	Aβ42 (H4/sw) (% from vehicle-treated)							Aβ42 (N2a/sw) (% from vehicle-treated)					
-10.505						97.4							
-9.89791	80.5	84.4		96.1	102	99.6	100	105.6	89.8	140.1	145.9	108.9	71.8
-9.39794	77.8	75.6	84.9	88	98.9	100.8	91.6	92.5	91.0	93.3	94.8	64.7	62.6
-8.89794	59.1	54.5	83.6	83.3	101.1	88	90.3	53.1	43.4	58.1	84.4	39.5	51.4
-8.39794	37.2	35.6	48.5	60.1	60.6	69.1	71.5	42.4	41.5	27.8	32.4	15.3	17.9
-7.89794	15.4	14.2	20.7	28.2	28.7	42.1	36.8	18.0	15.5	4.9	5.8	7.7	6.0
-7.39794	10.9	10.1	12.1	8.9	6.2	12.3	9.7	7.9	8.7	2.1	3.4	3.8	5.7
-6.89794	3	2.7	3.8	2.7	1.5	3.3	3.1	5.7	5.2	3.4	4.6	3.8	2.5
-6.39794	1.4	1.3	1	1.2	0.6		1.2	3.3	4.5	3.4	4.6	2.6	4.1

RO7019009 [lg(M)]	Aβ42 (HEK293/sw) (% from vehicle-treated)		
-10.3345	91.3	100	102.7
-9.5563	90.9	95.5	101.3
-8.77815	83.9	82.5	96.4
-8	54.7	49.3	67.5
-7.22185	19.2	20.6	27.7
-6.4437	2.1	n.d.	n.d.

RO7019009 [lg(M)]	Notch1 (% from vehicle-treated)	
-8.5	103.6	102.9
-8	103.7	107.5
-7.5	97.1	114
-7	101.1	101.2
-6.5	94.9	98.8
-6	101.1	95.6
-5.5	104.8	95.9
-5	94.2	83.6

E

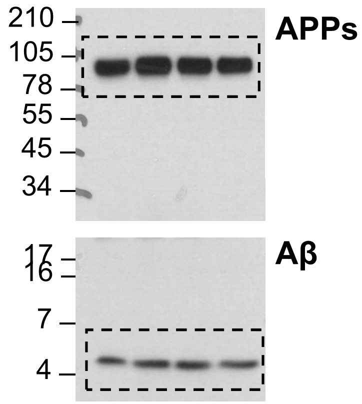

Aβ/APPs (Rel. to DMSO-treated)

RO7019009		
DMSO	1	1
50 nM	0.954	1.080
250 nM	1.029	0.886
500 nM	0.860	0.901

F

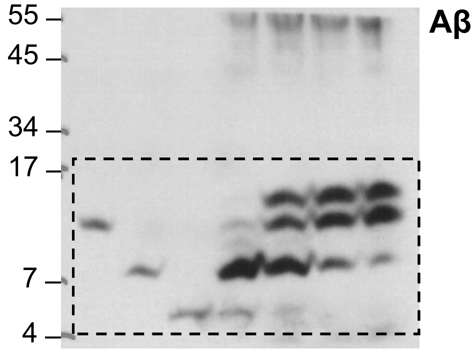

Source Data Figure 3
