# Supplementary material for: Aβ43‐producing PS1 FAD mutants cause altered substrate interactions and respond to γ‐secretase modulation
Source: EMBO Rep. 2019 Nov 25;21(1):e47996. doi: 10.15252/embr.201947996 (PMC6945062; doi:10.15252/embr.201947996)
Supplement: Supplementary file 9 — Source Data for Figure 4 [file EMBR-21-e47996-s007.pdf]

A Aβ38 (% of total Aβ)

	DMSO					50 nM RO7019009					250 nM RO7019009					500 nM RO7019009				
WT	9.8	11.4	12.6	4.8	4.3	30.5	42.9	35.3	37	33.6	65.1	67.3	70.2	70.5	53.7	93.2	79.1	78.7	82.6	77.8
M292D	7.4	29.8	8.8	2.1	2.1	32.5	24	27	22.5	20.3	47.8	51.8	61.2	59.2	54.4	74.4	66.6	74.1	72.7	67.5
V261F	15	n.d.	n.d.	1.2	1	33.5	34	25.1	16	18.1	60.7	54	41.1	47.4	43.6	68	55.7	51.7	63.8	60.4
R278I	n.d.	n.d.	n.d.	1.1	0.2	22.2	19.7	18.4	22.1	20.5	47.5	48	44.2	53.1	53.9	58.7	63.1	59.9	68.2	66.4
L435F	7.3	9.4	10.1	3.9	3.9	32.7	36	39	44	44.4	67	73.1	68.5	67.2	77.7	74.9	83.2	77.7	82.6	84.2
L166P	5.1	n.d.	n.d.	0.9	1	14.1	19.9	17.7	20.2	18.9	33.6	42.7	38.6	41.1	33.3	62.1	51.8	51.9	48.5	47.4
Y256S	20.4	18	16.1	1.5	15.3	78.4	78.7	80.1	74.5	77.6	88.8	91.4	91.2	88.7	89.2	89.6	92	94.1	92.4	93.1
G382A	29.6	23.6	23.2	12.2	12.2	90.2	85.3	84.8	85.7	88	95.7	95	94.6	94.3	94.4	93.4	98.1	95.1	94.3	95.3

B Aβ43 (% of total Aβ)

	DMSO					50 nM RO7019009					250 nM RO7019009					500 nM RO7019009				
WT	n.d.	0.5	0.5	0	0.2	n.d.	0.9	0.9	0	0.3	n.d.	1.2	1	n.d.	0.3	n.d.	0.9	1	n.d.	0.2
M292D	2.2	1.9	2.5	3.6	4.1	4.2	2	2.2	2.4	3	n.d.	2.3	1.5	1.3	1.7	n.d.	2.7	0.9	0.8	0.6
V261F	47.7	59.1	57.1	68.3	69.2	49.7	44.3	49.3	66.2	64.4	29.8	36.5	41	41.9	46.2	25.4	33.7	33.6	28.5	32.8
R278I	60.9	68.3	68.8	68.5	71	46.8	57.8	56.5	55.3	59.9	34.6	34.5	33.6	30.3	31.4	20.5	20.6	22.3	17.8	20.4
L435F	18.6	21.6	17.9	23.3	32	21.5	19.3	19.2	21.4	25.1	13.1	9.5	12.2	18.9	11.9	8.9	5.2	11.7	7.3	8.5
L166P	19	30.1	32.9	26.6	35.5	17.3	23.6	23.3	20	22.1	10	14.2	13	10.9	14.7	6.5	9.6	7.5	6.6	8.8
Y256S	16.5	21.4	19.2	21.1	19.6	7.6	8	5.9	6.6	6.9	4.3	3.5	2.6	2.2	3.4	3.7	2.7	n.d.	0.8	1.9
G382A	15.7	11.2	11.8	10.6	11.6	2.9	2.5	2.1	1.8	1.9	1.6	1.1	0.7	0.4	0.7	1.6	1.5	n.d.	0.2	0.5

C Aβ42 (% of total Aβ)

	DMSO					50 nM RO7019009					250 nM RO7019009					500 nM RO7019009				
WT	4.9	4.2	3.3	6.4	3.7	3	2.6	2.1	2.6	1.8	2.5	1.6	1.7	1	1	n.d.	1.2	1.2	0.6	0.5
M292D	6.8	2.6	4.8	8.1	6	6.4	2.5	4.7	3.4	3.1	3.2	2.1	1.7	2.1	1.7	3.9	1.9	1.3	2.2	1.5
V261F	5.1	4.6	7.3	5.6	5.5	4.5	2.7	5.4	5.5	5.8	4.5	3.3	4.7	5.2	4.8	4	2.8	5.3	4.3	3.6
R278I	13.5	9.4	12	10.9	9.1	12.5	9.3	11.6	10.1	7.3	14.1	8	10.8	8.5	7.7	10.6	6.4	9.2	7	6.5
L435F	5.3	4.3	4	4.5	3.6	3.9	1.9	2.6	2.3	1.8	3.2	1.1	2	1.3	1	2.8	n.d.	1.6	0.8	1
L166P	27.7	25.9	31.6	32.6	24.5	32.5	26.3	31.9	34.1	29.3	28.7	25.8	30.8	28.8	31.7	23	23.3	26.6	28.4	26.7
Y256S	23.5	22.3	25.6	29.5	21.5	6.3	5.5	6.5	7.7	5.2	4.6	2.6	3.3	4.3	3.8	4.3	2.2	4	2.8	2.7
G382A	8.7	3.3	4.4	4.4	3.4	1.6	0.6	1.2	0.6	0.5	1	0.5	0.8	0.2	0.2	1.2	0.4	1.4	0.4	0.2

Source Data Figure 4
