# Supplementary material for: Aβ43‐producing PS1 FAD mutants cause altered substrate interactions and respond to γ‐secretase modulation
Source: EMBO Rep. 2019 Nov 25;21(1):e47996. doi: 10.15252/embr.201947996 (PMC6945062; doi:10.15252/embr.201947996)
Supplement: Supplementary file 10 — Source Data for Figure 6 [file EMBR-21-e47996-s008.pdf]

A

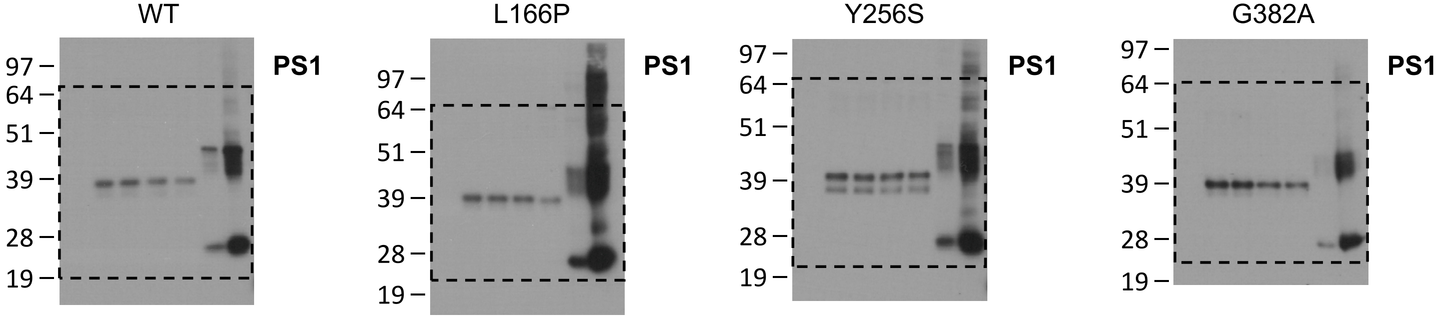

B V44 CL efficiency (Rel. to DMSO-treated)

	DMSO										
WT	1	1	1	1	1	1	1	1	1	1	1
L166P					1	1	1	1	1	1	1
Y256S				1	1	1		1	1	1	1
G382A	1	1	1		1	1		1	1	1	1

	50 nM RO7019009										
WT	0.89	0.87	0.99	1.03	0.92	0.78	1.05	1.03	1.06	1.1	1.45
L166P					1.04	0.89	1.17	1	0.74	1.19	1.11
Y256S				1.14	1.15	0.93		0.94	1.05	1.06	1.15
G382A	0.89	0.98	1.06		1.29	1.22		0.94	0.64	0.79	0.93

	500 nM RO7019009										
WT	0.76	0.83	0.8	0.83	0.7	0.41	0.82	0.85	1	1.02	1.37
L166P					1.16	0.75	0.62	1.04	0.92	1.24	0.74
Y256S				0.77	1.06	0.87		1.03	0.76	1.14	0.86
G382A	0.74	1.11	0.83		1.06	0.89		0.91	0.76	0.81	0.67

	2500 nM RO7019009										
WT	0.61	0.45	0.49	0.39	0.4	0.26	0.48	0.66	0.79	0.58	0.58
L166P					0.87	1.01	0.75	0.73	0.58	0.86	0.43
Y256S				0.83	1.26	1.1		1.11	0.8	1.1	0.68
G382A	0.6	0.91	0.78		1	0.49		0.9	0.52	0.66	0.59

C

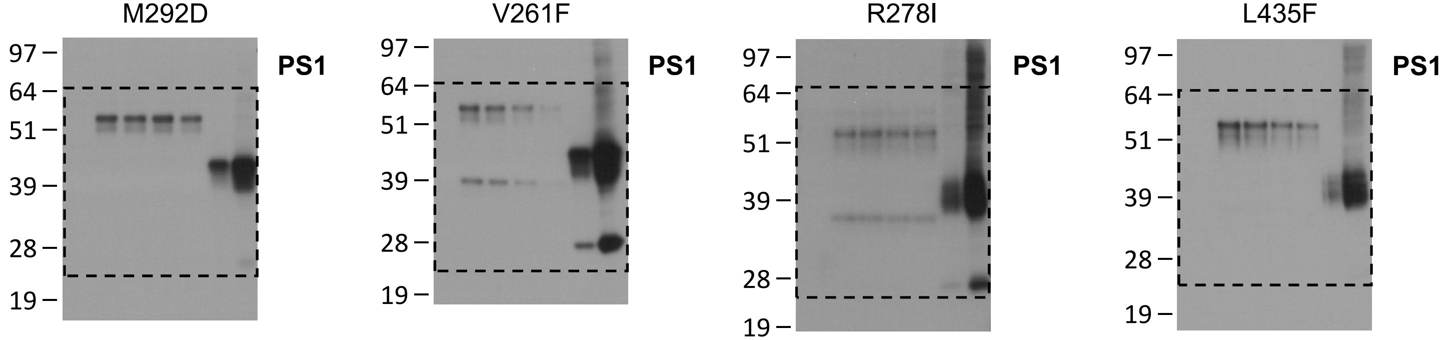

D V44 CL efficiency (Rel. to DMSO-treated)

	DMSO								
M292D	1	1	1	1	1	1	1	1	1
V261F			1	1	1	1	1	1	1
R278I		1	1		1	1	1	1	1
L435F	1		1	1	1	1	1	1	1

	50 nM RO7019009								
M292D	1.08	1.28	1.08	1.22	1.32	0.95	0.57	1.17	0.78
V261F			0.89	0.8	0.99	0.82	0.62	1	0.77
R278I		1.34	0.96		1.4	1.02	0.59	1.16	0.83
L435F	1		0.7	1.05	0.85	0.94	1.24	0.85	1.13

	500 nM RO7019009								
M292D	0.97	0.95	0.8	1.01	0.74	1.04	0.65	1.1	0.61
V261F			0.71	0.59	0.85	0.55	0.49	0.66	0.86
R278I		1.46	0.99		1.43	1.01	0.59	1.06	0.61
L435F	1.06		0.58	0.73	0.62	0.67	0.75	0.63	0.93

	2500 nM RO7019009								
M292D	0.88	0.99	0.88	0.64	0.65	0.81	0.43	0.55	0.38
V261F			0.57	0.55	0.72	0.3	0.76	0.54	0.68
R278I		1.23	0.76		1.18	0.92	0.3	1.06	0.85
L435F	1.08		0.52	0.42	0.6	0.71	0.75	0.58	0.47

Source Data Figure 6
